# Traumatic cataract in patient with anterior megalophthalmos: Case report

**DOI:** 10.1097/MD.0000000000007160

**Published:** 2017-07-28

**Authors:** Edyta Chlasta-Twardzik, Anna Nowińska, Paweł Wąs, Agnieszka Jakubowska, Edward Wylęgała

**Affiliations:** Chair and Department of Ophthalmology, School of Medicine with the Division of Dentistry in Zabrze, Medical University of Silesia, Katowice, Poland.

**Keywords:** AMD, anti-VEGF, cataract surgery, CNV, diabetic retinopathy, megalocornea, megalophthalmos anterior, Optical Coherence Tomography, Optical Coherence Tomography Angiography (OCT-A), traumatic cataract

## Abstract

**Rationale:**

Megalophthalmos anterior is a rare, bilateral, nonprogressive, hereditary, congenital disorder, characterized by the enlargement of all anterior segment structures of the eye, with megalocornea, iris atrophy, and zonular abnormalities commonly found. Usually almost asymptomatic in young patients, with most complaints concerning blurred vision due to the common corneal astigmatism, it might in time lead to several complications including premature cataract formation and pigmentary glaucoma.

**Patient concerns:**

This review presents the case of a 47-year old patient referred to our clinic for traumatic cataract surgery, with striking bilateral megalocornea, somehow overlooked during previous ophthalmic examinations in his local outpatient clinic.

**Diagnosis:**

We noticed markedly enlarged corneas and deepened anterior chambers of his both eyes, accompanied by intumescent, white cataract of the right eye, and incipient cortical cataract of the left eye. Best corrected visual acuity (BCVA) was counting fingers in the right eye and 20/25 in the left eye. Additional examination revealed multiple abnormalities of the anterior segment, leading to the diagnosis of anterior megalophthalmos. It is astounding the patient remained undiagnosed during previous examinations, with his megalocornea and remarkably deep anterior chamber so apparent.

**Interventions and outcomes:**

We performed standard phacoemulsification procedure, with 3 piece posterior chamber intraocular lens (PCIOL) implantation into the lens capsule. The surgery was uneventful, with postoperative BCVA of 20/20 in the right eye, and no dislocation of the lens in 9-month observation period.

**Lessons:**

Complicated cataract in patients with anterior megalophthalmos can be successfully treated with standard phacoemulsification procedure followed by the bag PCIOL implantation.Care needs to be taken during cataract surgery in these patients, as zonular abnormalities and lens enlargement are common, resulting in increased rate of intra- and postoperative complications. As patients with anterior megalophthalmos require a more careful follow-up.

## 1 Introduction

Anterior megalophthalmos, first described in 1914, as cited by Wright^[1]^ is a rare nonprogressive developmental disorder of the eye characterized by megalocornea, deepened anterior chamber, and Zinn membrane abnormalities.^[2]^ In isolated megalocornea, corneal diameter is enlarged to 13 mm or more, with normal corneal histological structure, normal, or mildly decreased corneal thickness and normal curvatures. With-the-rule astigmatism is a common symptom.^[2]^ In anterior megalophthalmos, megalocornea is accompanied by retroposition of the iridolenticular diaphragm with a deepened anterior chamber, enlarged ciliary ring with zonular abnormalities and iridodonesis, lens enlargement, and subluxation, with frequent premature cataract formation. The axial length of the vitreous chamber is thus reduced but the axial length of the eyeball usually remains normal.^[2,3]^

Other anterior segment abnormalities in anterior megalophthalmos include central mosaic corneal dystrophy, iris hypoplasia, iridocorneal angle dysgenesis, pigmentary dispersion syndrome with Krukenberg spindle and pigmentary glaucoma.^[2,3]^

Posterior segment disorders described in anterior megalophthalmos include vitreous fibrillar degeneration, peripherial retinal degeneration, breaks, and neovascularisation, sometimes leading to retinal detachment or vitreous haemorrhage.^[4,5]^ Visual acuity is usually good.^[2]^ Abnormal eye cup closure during the developmental process is considered an ethiological factor of the disease. As reported in most cases (90%), megalophthalmos anterior is an X-linked recessive condition more common among males.^[2,3]^ Cases of autosomal (dominant and recessive) inheritance as well as sporadic cases have also been described.^[2]^

The differential diagnosis of anterior megalophthalmos includes megalocornea, congenital glaucoma, and keratoglobus.^[6]^

Congenital glaucoma is a progressive and often asymmetrical disease, with symptoms including tearing and photophobia, characterized by increased intraocular pressure (IOP), Descemet membrane ruptures (Haab striae), endothelial cells disorders, increased axial length of the eye, and optic nerve atrophy. Keratoglobus is a corneal ectasia with normal or slightly increased corneal diameter, significant thinning, and abnormal curvatures.

## 2 Purpose

The aim of the case report is to present traumatic cataract management in a patient with this rare condition.

## 3 Case report

**Figure 1:**
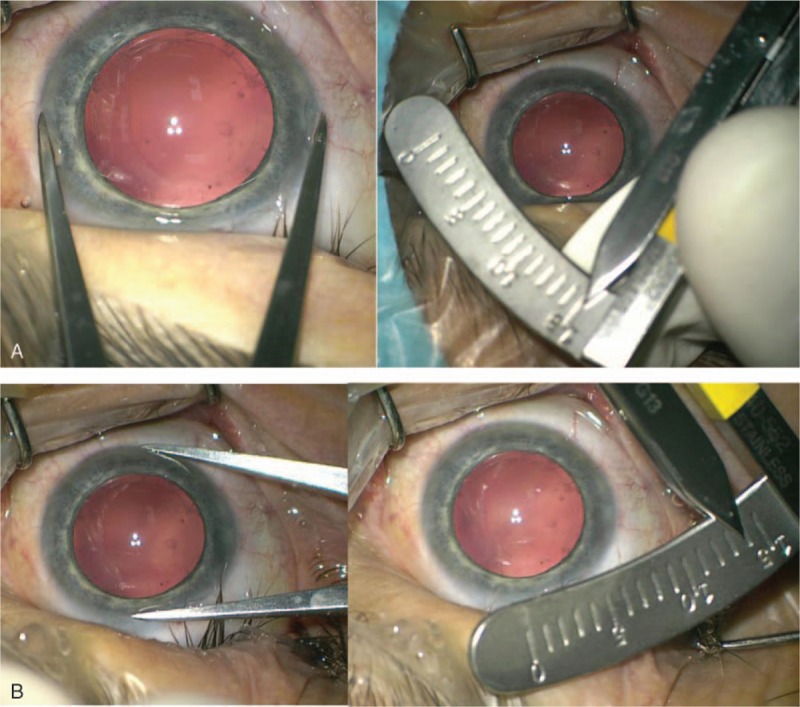
A and B, Color photography of the right eye. The corneal diameter was measured with paired calipers. Significant enlargement of the cornea to 14 × 15 mm.

**Figure 2:**
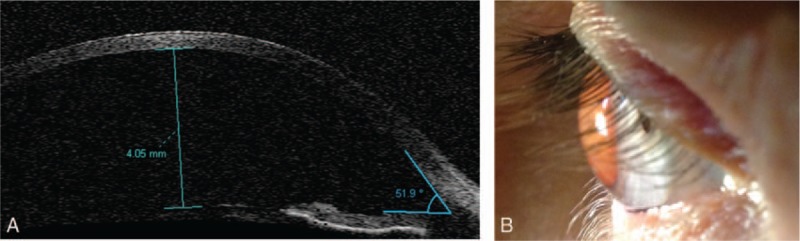
A, Time domain anterior segment OCT. Anterior chamber single scan of the right eye. Significantly increased anterior eye chamber depth of 4.05 mm. Very wide iridocorneal angle of 51.9^∗^. B, Color photography of the right eye in the slit-lamp microscopy examination showed significantly increased anterior eye chamber depth. OCT = Optical Coherence Tomography.

**Figure 3:**
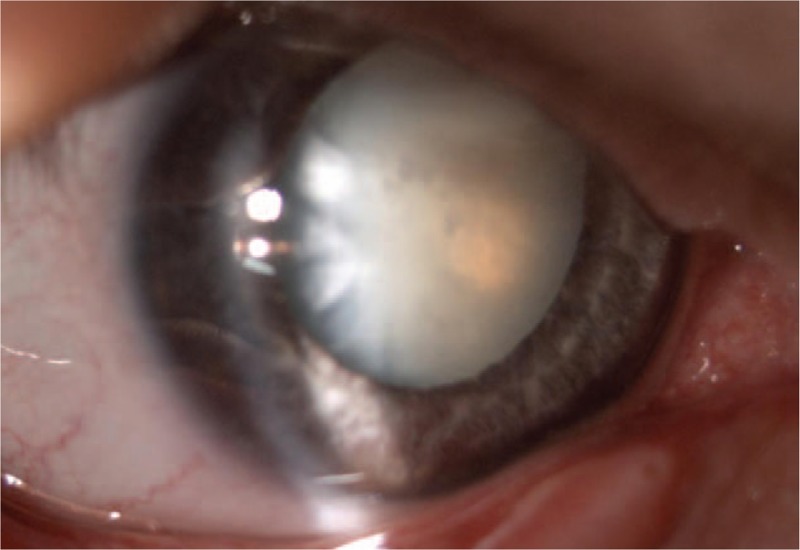
Color photography of the right eye in the slit-lamp microscopy examination showing mature intumescent cataract.

A 47-year-old man was urgently admitted to Clinical Department of Ophthalmology, School of Medicine with the Division of Dentistry in Zabrze, Medical University of Silesia in Katowice for cataract surgery. The patient reported a blunt-force trauma to the eye followed by a progressive visual acuity deterioration. Shortly after the injury, the patient underwent eye examination at a local clinic due to progressive visual acuity deterioration. The presence of intraocular foreign body was excluded. The patient had previously spectacle correction of hypermetropic astigmatism, but had never been diagnosed for other ocular diseases or received ophthalmological treatment. Examination on admission revealed bilateral, symmetrical enlargement of the cornea (right/left eye 14 × 15 mm) (Fig. 1A and B), deepened anterior chamber (Fig. 2A and B), hypoplasia of the iris with minor transillumination defects and iridodonesis, as well as mature intumescent cataract of the right eye (Fig. 3) and minor cortical and posterior capsule opacifications of the left eye.

Best corrected visual acuity (BCVA): right eye—counting fingers with full light projection; left eye (cc +4.0Dsph –2.0Dcyl. ax 180^∗^) 20/25. IOP—right eye 12 mm Hg; left eye 13 mm Hg. Axial length—right eye (A-scan ultrasound biometry): 22.90 mm; left eye (IOL Master Carl Zeiss Meditec Inc, Dublin, CA): 23.40 mm.

**Figure 4:**
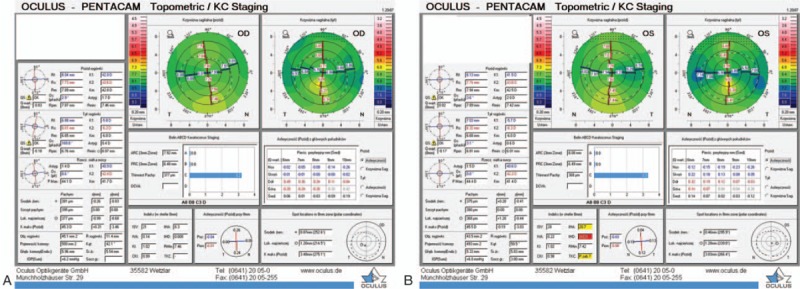
A and B, Corneal topography of the right eye. Central keratometry (front) is this: right eye 42.0/43.6D; left eye 41.5/43.6D. The corneal maps reveal with the rule corneal astigmatism and decreased corneal thickness with no signs of keratoconus.

Keratometry (The *Pentacam HR*, *Type 70900*; Oculus Germany) right eye: 42.0/43.6D; left eye 41.5/43.6D, revealed with the rule corneal astigmatism, with corneal thinning with no signs of keratoconus (Fig. 4A and B).

Pachymetry (The *Pentacam HR*, *Type 70900*; Oculus Germany): right eye: 395 um; left eye: 380 um.

Corneal endothelial cell density (Topcon SP-3000P) right eye: 2354/mm^2^; left eye 2323/mm^2^.

Anterior segment Optical Coherence Tomography (OCT) (OCT Visante Carl Zeiss Meditec Inc, Dublin, CA) revealed wide, open iridocorneal angles, the anterior chamber depth was 4.05 mm in the right eye and 4.82 mm in the left eye. It was impossible to obtain scans of the whole anterior chamber due to significant enlargement of the anterior part.

**Figure 5:**
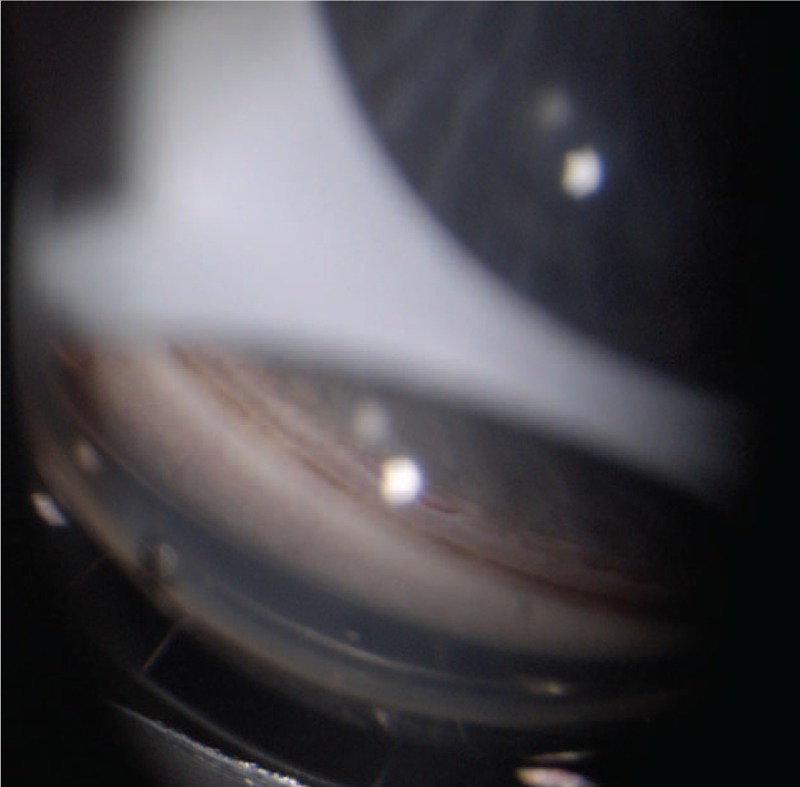
Color photography of the right eye in gonioscopy examination. Very deep and wide iridocorneal angle, with marked displacement of the iridolenticular diaphragm and trabeculum posteriorly from the Schwalbe line. Significant pigmentation of the trabecular meshwork.

Gonioscopy revealed a wide, very deep iridocorneal angle with a significant displacement of the iridolenticular diaphragm and trabeculum posteriorly from the Schwalbe line, as well as significant pigmentation of the trabecular meshwork (Fig. 5).

Fundus examination of the left eye was irrelevant. Notice that despite the marked pigmentation of the trabecular meshwork, the IOP was normal and no signs of glaucoma were found on fundoscopy; the fundus of the right eye was not assessable on admission.

Based on medical history and the clinical picture, complicated intumescent cataract of the right eye, initial cortical and posterior capsular cataract of the left eye as well as bilateral megalophthalmos anterior were diagnosed.

**Figure 6:**
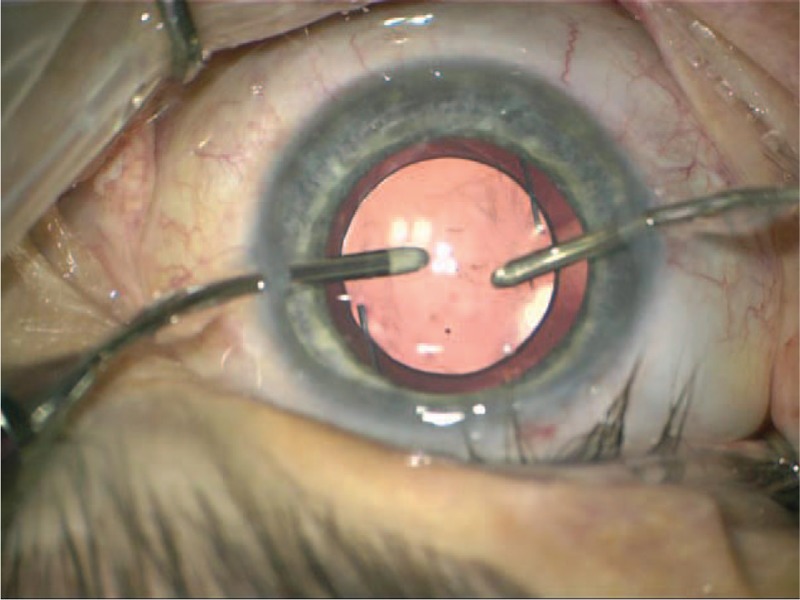
Color photography of the right eye. Small incision cataract surgery. Intraocular lens implanted into the lens capsule.

After a qualifying examination and consultation with an anesthetist, the patient was qualified for a surgery under local anesthesia the following day. A typical small incision phacoemulsification procedure with implantation of the 3-piece posterior chamber intraocular lens (PCIOL) (Alcon AcrySof MN60AC) into the lens capsule was performed (Fig. 6). The surgery was uncomplicated. Minor lens subluxation was found intraoperatively. The patient was discharged home on the first day after surgery in good overall and local condition and with appropriate instructions. He reported for a follow-up 7 days after hospital discharge.

Postoperative follow-up:

- autorefractometry of the right and the left eye: +2.25D −1.0 Dcyl 180^∗^; +4.0 Dsph −2.0 Dcyl ax180^∗^, respectively.
- the best corrected visual acuity: 20/30 cc +2.25 Dsph – 1.0 D. cyl. ax 180^∗^ in the right eye; 20/25 cc +4,0Dsph −2,0 Dcyl ax 180^∗^ in the left eye.

The anterior segment of the right eye was stable with transparent optical media, proper centration of the intraocular lens, and slight iridodonesis. The fundus image of the right eye was normal. Again, despite the marked pigmentation of the trabecular meshwork, the IOP was normal; no signs of glaucoma were found in fundoscopy in the operated eye as well.

In a follow-up examination 9 months after the surgery:

- the best corrected visual acuity: 20/25 sc; 20/20 cc +0,75 Dsph in the right eye; 20/200 sc, 20/100 cc +2,5 Dsph −0,75 Dcyl ax 180^∗^ in the left eye.
- intraocular pressure: right eye 14 mm Hg; left eye 14 mm Hg.

## 4 Discussion

Megalophthalmos anterior is a rare, nonprogressive hereditary condition, frequently associated with cataract formation in young patients (ages 30–50). Owing to the presence of malformations in the anterior segment of the eyeball, particularly the ciliary zonules, lens capsule, and the iris, surgical treatment of cataract in these patients may be associated with complications both of the intraoperative (damage rendered to the capsule or the suspensory ligament of the lens accompanied by vitreous loss)^[7,8]^ and postoperative (lens dislocation, retinal detachment) course^[7–9]^ with the complication rate having substantially decreased with the advancement of surgical techniques.^[7]^

Due to the enlarged diameter of the lens capsule, PCIOL implanted in the capsular bag have been described to dislocate. To prevent this complication in megalophthalmos anterior patients, an anterior chamber intra ocular lens (IOL) has been employed by some authors.^[6,9]^ Also, using larger length IOL (16–18 mm) has been postulated.^[9]^

On the other hand, some authors ^[8,10–12]^ have been able to successfully use a 13.5 mm polimetylometakrylan posterior chamber intraocular lens (PMMA PCIOL) in patients with megalophthalmos anterior, de Sanctis and Grignolo^[13]^ —a single piece foldable lens, while Ehud et al^[14]^ and Zare et al^[15]^ successfully implanted a foldable Alcon MN60AC lens in 3 operated eyes with anterior megalophthalmos.

In their work, Zare et al used ultrasound biomicroscopy (UBM) for the preoperative measurement of the lens capsule to estimate the risk of IOL dislocation after the surgery.

In our patient, we have performed standard small incision phacoemulsification cataract surgery, implanting a 3-piece Alcon AcrySof MN60AC PCIOL into the capsular bag, achieving proper centration of the implant, with slight phacodenesis and satisfactory postoperative corrected visual aquity. The SRK/T formula was used to calculate the IOL power.

**Figure 7:**
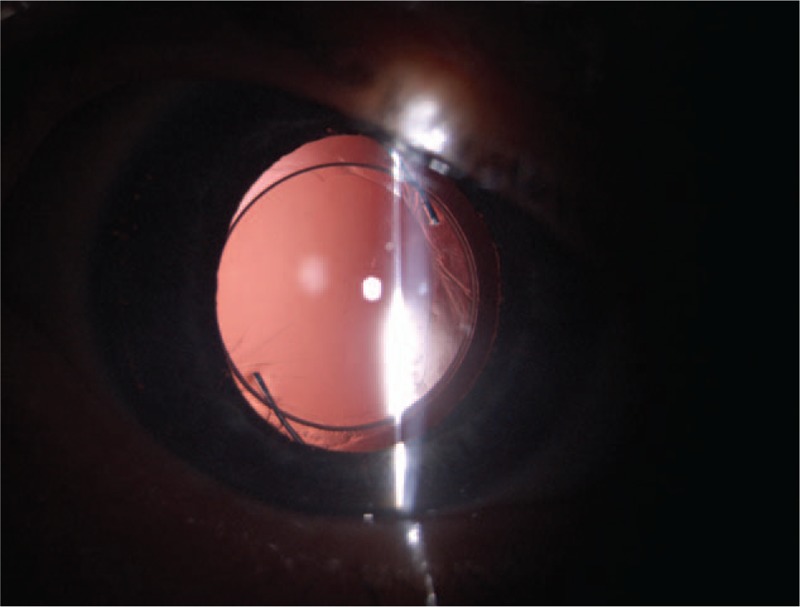
Color photography of the right eye 6 months after surgery, in the slit lamp microscopy examination, showing the implant in an axial position, within the capsular bag transillumination defects of the peripherial iris.

In a follow-up examination 9 months after the surgery, the implant was found to remain in an axial position, within the capsular bag, and the slight phacodenesis had not deteriorated (Fig. 7).

Postoperative refractometry revealed postoperative hyperopia, possibly due to the presence of a relative shift of the lens-iris diaphragm toward the back and decreased vitreous axial length in the course of the described condition. Our outcome is similar to the ones reported by Ehud et al^[14]^ and Zare et al.^[15]^

## 5 Conclusion

To conclude, it is worth noticing that due to mild subjective symptoms of anterior megalophthalmos, it is a condition that might be easily overlooked during standard, routine ophthalmic examination. A potentially dangerous omission, as patients with anterior megalophthalmos require more frequent and careful follow-up due to the possible complications.

With premature cataract formation being one of the most common complications, patients with anterior megalophthalmos will likely require cataract surgery relatively early, and due to the abnormalities of Zinn's membrane, the surgery might be complicated. However, despite the malformations in the anterior segment of the eye, cataract phacoemulsification with PCIOL implantation into the capsular bag may be successfully performed in megalophthalmos anterior patients.

As the structures of the anterior segment are enlarged, applying increased IOL length may help to reduce the risk of IOL dislocation in the postoperative period, yet having used a standard 13-mm Alcon MN60AC IOL, we, like Assia et al and Zare et al before, observed no dislocation in a 6-month follow-up examination.

Preoperative UBM examination might be useful to exclude patients with significantly enlarged lens, having high risk of IOL dislocation.

The increased anterior chamber depth accompanied by the reduced vitreous axial length may affect the postoperative refraction results, and the probability of postoperative hyperopia should be taken into consideration during IOL calculation in these patients.
